# Glycemic Control and Metabolic Parameters in Children and Adolescents With Type 1 Diabetes

**DOI:** 10.7759/cureus.43416

**Published:** 2023-08-13

**Authors:** Marta Canha, Sofia Ferreira, Rita Santos Silva, Aida Azevedo, Ana S Rodrigues, Cintia Castro-Correia

**Affiliations:** 1 Endocrinology, Diabetes and Metabolism, Centro Hospitalar Universitário São joão, Porto, PRT; 2 Pediatric Endocrinology and Diabetology Unit, Centro Hospitalar Universitário São joão, Porto, PRT; 3 Pediatrics, Centro Hospitalar do Médio Ave, Vila Nova de Famalicão, PRT; 4 Paediatrics, Centro Hospitalar do Médio Ave, Vila Nova de Famalicão, PRT

**Keywords:** metabolic control, a1c, time-in-range, children and adolescents, type 1 diabetes

## Abstract

Aim: The association between glycemic control and metabolic status is poorly defined in children and adolescents with T1D, besides being biologically plausible. We aimed to evaluate the association between glycemic control and body mass index (BMI), blood pressure (BP), and lipid profile in children and adolescents with T1D.

Methods: Observational cross-sectional study including children and adolescents (5-18 years old) followed in our outpatient clinic with the diagnosis of T1D for at least a year. We used linear regression models (unadjusted and adjusted to sex and age) to evaluate the association between glycated hemoglobin (A1c) and time in range (TIR), several prespecified metabolic parameters, and prespecified demographic and clinical characteristics. We considered a p-value of <0.05 to be statistically significant.

Results: A total of 144 patients were included, 51% of whom were female. The population had a mean age of 12.7±3.4 years old. We report a positive association between A1c and BMI, systolic and diastolic BP, total- and LDL-cholesterol and triglycerides. Females and patients diagnosed at a younger age presented with higher A1c values. There is a tendency for a negative association between TIR and the former parameters. Higher A1c levels and lower TIR were associated with higher glycemic variability and were treated with a higher basal insulin per Kg dose.

Conclusion: Our results support an important association between worse glycemic control and an unhealthier metabolic profile in children and adolescents with T1D. We can hypothesize that a good glycemic profile is needed to achieve good metabolic control at a young age.

## Introduction

Treatment of type 1 diabetes mellitus (T1DM) in children and adolescents is challenging, and only a small percentage of patients achieve the recommended targets of glycemic control. [1] The evaluation of glycemic control solely depended on glycated hemoglobin (A1c) for a long time. Nowadays, continuous glucose monitoring (CGM) has changed this paradigm, and time in range (TIR) has been largely used for that purpose [1].

The American Diabetes Association guidelines for youth with T1DM highlight the need for the management of lipids, blood pressure, and weight, as well as the specific demand for glycemic control [2]. The association between glycemic control and metabolic status is poorly investigated in children and adolescents with T1DM beyond being biologically plausible.

For instance, higher body weight in T1DM may lead to insulin resistance and poorer glycaemic control. On the other hand, being underweight may denote unhealthy growth and development [3,4]. Existing data hints that better glucose control is associated with a more favorable lipid profile [5]. Also, the lipid profile is believed to be crucial regarding cardiometabolic risk. Both hyperglycemia and dyslipidemia are common in patients with T1DM, and both increase cardiovascular disease risk [6]. Finally, blood pressure is thought to be an important predictor of retinopathy and nephropathy in young patients with T1DM [7].

Given the paucity of data, we aimed to evaluate the association between glycemic control (A1c and TIR) and body mass index (BMI), blood pressure (BP), and lipid profile in children and adolescents with T1DM.

## Materials and methods

This study was reviewed and approved by the ethical committee of our center. Written informed consent for participation was not required for this study in accordance with national legislation and institutional requirements. The privacy of the patients included was preserved throughout the study. 

Study design and study participants

This is a cross-sectional observational study evaluating children and adolescents (5-18 years old) followed in an outpatient clinic in our tertiary center with the diagnosis of T1DM for over a year. 

Data collection and parameters definitions

The following demographic parameters were collected from medical records: sex, age at diagnosis, and age at evaluation. At the time-point evaluation, the following clinical parameters were collected: weight, height, systolic BP, diastolic BP, A1c, lipidic profile (total cholesterol, LDL cholesterol, HDL cholesterol, and triglycerides), total daily basal insulin, delivery method (continuous subcutaneous insulin infusion, CSII, or multiple daily injections).

CGM-based glucose metrics were collected whenever they became available from LibreView within the 30 days before the appointment. These metrics were defined considering ATTD consensus recommendations [8]:

TIR: CGM-based percentage of time spent in the target range of 70-180 mg/dL;

Time above range, TAR: percentage of time spent >180mg/dL;

Time below range, TBR: percentage of time spent <70mg/dL. 

Outcomes and statistical analysis

Continuous variables are described as mean ± standard deviation, or median (25th to 75th percentiles), and categorical variables as proportions (percentages). We performed unadjusted and adjusted linear regression analyses to evaluate the association between A1c and TIR and several prespecified metabolic parameters. The adjusted model included sex and age. We also performed unadjusted and adjusted linear regression analyses to evaluate the association between A1c and TIR and the clinical and demographic characteristics of patients. Statistical analyses were conducted using Stata software, version 14.1 (StataCorp). We considered a two-sided p-value of less than 0.05 to be statistically significant.

## Results

Baseline population characteristics

Table 1 shows the demographic and clinical characteristics of the included population (n=144). The population had a mean age of 12.7±3.4 years old, with 51% being female. The median BMI percentile was 20.4±3.7kg/m2. The average A1c was 8.1±1.2%, and the TIR was 47.7±17.3%.

**Table 1:** Clinical and demographic characteristics of the population included (n=144). BMI: Body mass index; A1c: Glycated hemoglobin; CSII: Continuous subcutaneous insulin infusion; TIR: Time in range; TAR: Time above range; TBR: Time below range. Values are shown as mean ± standard deviation or as median [percentile 25 to percentile 75].

Feminine sex, n (%)	73 (50.7)
Age at diagnosis, years	6.7 ± 3.7
Age at evaluation, years	12.7 ± 3.4
Weight, kg	49.3 ± 16.2
Height, cm	153.0 ± 17.2
BMI, kg/m^2^	20.4 ± 3.7
BMI percentile	67.4 [46.2; 83.0]
Systolic blood pressure, mmHg	112.5 ± 11.0
Diastolic blood pressure, mmHg	65.0 ± 8.8
A1c, %	8.1 ± 1.2
Total cholesterol, mg/dL	159.0 ± 31.4
HDL cholesterol, mg/dL	56.6 ± 9.5
LDL cholesterol, mg/dL	89.8 ± 27.6
Triglycerides, mg/dL	63.1 ± 29.3
CSII users, n (%)	122 (85.9)
Continuous Glucose Monitoring users, n (%)	128 (88.9)
Total daily basal insulin, UI	20.2 ± 8.6
Total daily basal insulin per Kg, UI/kg	0.4 ± 0.1
TIR, %	47.7 ± 17.3
TAR, %	48.6 ± 18.0
TBR, %	3.6 ± 4.2
Coefficient of variation, %	39.5 ± 7.0

Association between A1c and TIR and metabolic parameters

Table 2 regards the association between A1c, TIR, and metabolic parameters. There was a positive association between A1c and BMI, systolic and diastolic BP, total and LDL cholesterol, and triglycerides. These associations remain significant after adjusting for sex and age at evaluation. Although not statistically significant (in a possible association with a lack of power given the missing data concerning TIR as expressed in Table 4), there is a tendency for a negative association between TIR and the former parameters.

**Table 2:** Association between A1c and TIR and metabolic parameters. BMI: Body mass index; A1c: Glycated hemoglobin; TIR: Time in range. † Adjusted for sex and age at evaluation.

	A1c		TIR	
	b	p-value	b	p-value
BMI, kg/m^2^				
Non-adjusted	0.08	0.004	0.08	0.909
Adjusted model^†^	0.07	0.033	-0.55	0.537
Systolic blood pressure, mmHg				
Non-adjusted	0.02	0.010	-0.12	0.515
Adjusted model^†^	0.03	0.021	-0.24	0.233
Diastolic blood pressure, mmHg				
Non-adjusted	0.04	0.000	-0.26	0.245
Adjusted model^†^	0.04	0.001	-0.29	0.273
Total cholesterol, mg/dL				
Non-adjusted	0.01	0.000	-0.08	0.362
Adjusted model^†^	0.01	0.000	-0.09	0.321
HDL cholesterol, mg/dL				
Non-adjusted	0.002	0.882	-0.14	0.474
Adjusted model^†^	0.005	0.687	-0.11	0.585
LDL cholesterol, mg/dL				
Non-adjusted	0.02	0.000	-0.01	0.926
Adjusted model^†^	0.02	0.000	-0.03	0.811
Triglycerides, mg/dL				
Non-adjusted	0.01	0.004	-0.09	0.300
Adjusted model^†^	0.01	0.030	-0.11	0.202

Association between A1c and TIR and clinical and demographic characteristics

Table 3 shows the association between A1c and TIR and clinical and demographic characteristics. Patients diagnosed at an older age presented with lower A1c values and a higher TIR at CGM. Higher A1c levels and lower TIR were associated with higher TAB, TBR, and glycemic variability and were treated with a higher basal insulin per Kg dose.

**Table 3:** Association between A1c and TIR and clinical and demographic characteristics. A1c: Glycated hemoglobin; TIR: Time in range; TAR: Time above range; TBR: Time below range. * Codded as 1 if female and 0 if male. † Adjusted for sex and age at evaluation.

	A1c		TIR	
	b	p-value	b	p-value
Sex*				
Non-adjusted	0.42	0.036	0.79	0.839
Adjusted model^†^	0.38	0.059	0.95	0.809
Age at evaluation, years				
Non-adjusted	0.05	0.129	0.52	0.373
Adjusted model^†^	0.04	0.220	0.50	0.408
Age at diagnosis, years				
Non-adjusted	-0.04	0.194	1.43	0.003
Adjusted model^†^	-0.07	0.023	1.70	0.002
Total daily basal insulin, UI				
Non-adjusted	0.05	0.000	-0.39	0.110
Adjusted model^†^	0.05	0.001	-1.08	0.001
Total daily basal insulin per Kg, UI/kg				
Non-adjusted	3.74	0.000	-53.1	0.004
Adjusted model^†^	3.46	0.000	-61.4	0.001
TAR, %				
Non-adjusted	0.03	0.000	-0.93	0.000
Adjusted model^†^	0.03	0.000	-0.93	0.000
TBR, %				
Non-adjusted	0.01	0.764	-0.34	0.468
Adjusted model^†^	0.01	0.825	-0.32	0.486
Coefficient of variation, %				
Non-adjusted	0.06	0.003	-0.99	0.004
Adjusted model^†^	0.05	0.012	-0.85	0.019

Missing data per variable

Table 4 displays missing data per variable analyzed.

**Table 4:** Missing data per variable. BMI: Body mass index; A1c: Glycated hemoglobin; CSII: Continuous subcutaneous insulin infusion; TIR: Time in range; TAR: Time above range; TBR: Time below range.

Age at diagnosis, n (%)	5 (3.5)
Age at evaluation, n (%)	3 (2.1)
BMI, n (%)	2 (1.4)
Systolic blood pressure, n (%)	6 (4.2)
Diastolic blood pressure, n (%)	7 (4.9)
A1c, n (%)	2 (1.4)
Total cholesterol, n (%)	14 (9.7)
HDL cholesterol, n (%)	14 (9.7)
LDL cholesterol, n (%)	26 (18.1)
Triglycerides, n (%)	14 (9.7)
CSII users, n (%)	2 (1.4)
Total daily basal insulin, n (%)	9 (6.3)
TIR, n (%)	63 (43.8)
TAR, n (%)	64 (44.4)
TBR, n (%)	63 (43.8)
Coefficient of variation, n (%)	101 (70.1)

## Discussion

This is a cross-sectional study aiming to evaluate the association between glycemic control and several metabolic parameters in children and adolescents with T1DM. Our results show a significant positive association between A1c and BMI, systolic and diastolic BP, total and LDL-cholesterol, and triglycerides, and a tendency for a negative association between TIR and the former parameters. These results help to support the gap regarding this area of knowledge and suggest that in children and adolescents with T1DM, worse glycemic control is associated with an unhealthier metabolic profile. 

Here, we report a positive association between A1c and BMI and a negative tendency between TIR and BMI. The existing literature is not consensual on this topic. Data supporting the formerly known effect of increasing insulin to try to improve glycemic control, which contributes to increased BMI [9]. This led to recognizing the importance of minimizing weight gain while optimizing glycemic control to control cardiometabolic risk [9]. On the other hand, studies are agreeing with our results [10], and there is data stating no association between BMI and glycemic control [11].

We present an association between higher systolic and diastolic BP and higher A1c and a tendency between higher BP and lower TIR. Our results are in accordance with data from Chatterjee et al., who studied 24-hour ambulatory blood pressure in children and adolescents with type 1 diabetes mellitus. These authors concluded that poor diabetes control is associated with abnormal systolic BP [12]. Also, Shalaby et al. presented their findings in which higher A1c levels were associated with higher systolic and diastolic BP (diurnal and nocturnal) [13]. Nonetheless, there is data stating no associations between glycemic control and BP [14].

In our cohort, higher A1c was associated with higher levels of triglycerides and total and LDL cholesterol. Agreeing with our findings, Stankute et al. reported that A1c concentration was positively associated with levels of triglycerides, total- and LDL-cholesterol in a cohort of 883 patients under 25 years old [15]. Also, Shah et al. concluded that poor glycemic control predicted the likelihood of dyslipidemia and hypertriglyceridemia in poorly controlled Indian children with type 1 diabetes [16]. An analysis of the SEARCH study [17], including 1478 youth with type 1 diabetes, stated that worse glycemic control over time is a risk factor for the progression of dyslipidemia (progression meaning normal lipid concentrations at baseline and abnormal at follow-up) [18]. Contrarily, Mercedes Prado, et al. reported no significant differences concerning triglycerides, total-, LDL-, and HDL-cholesterol comparing children and adolescents with T1DM with A1c higher or lower than 7.5%; however, this was a small cohort, and the continuous analysis was not presented [19].

Considering the above, we can hypothesize that a good glycemic profile is needed to achieve good metabolic control at a young age and that this may reflect in future cardiometabolic risks. There is data on suboptimal diabetes control and abnormal microvasculature, a surrogate marker of damage to the cardiovascular system [20], which strengthens this hypothesis. It can also be hypothesized that the relationship might be bidirectional. 

Concerning our secondary analysis, we report that patients diagnosed at an older age presented with lower A1c values (a significant result for our adjusted model), in accordance with the results from Hashemipour et al. [21]. Also, patients who present higher A1c levels and lower TIR seem to be treated with a higher basal insulin per Kg dose; this is in accordance with previous data on the pediatric population [22-24]. It can be hypothesized that this may be driven by poorly compliant patients who are treated with high basal insulin, expecting it to reduce blood sugar levels. However, this may be deleterious. For instance, these patients may respond to reduced glucose levels with augmented food intake, which would induce further hyperglycemia [22]. 

There are limitations inherent to this study that must be acknowledged. First, this is a retrospective cross-sectional study, and, as such, we have considerable missing data regarding some important parameters such as TIR. Also, there are possible confounders that we may not have accounted for. Still, we believe that the importance of our results fairly overcomes these limitations and opens a door in this area of knowledge.

## Conclusions

Concluding, our results support an important association between worse glycemic control and an unhealthier metabolic profile in children and adolescents with T1DM. We can hypothesize that a good glycemic profile is needed to achieve good metabolic control at a young age and that this may reflect in future cardiometabolic risk. Prospective studies are needed to verify this hypothesis.
